# Subtype identification from heterogeneous TCGA datasets on a genomic scale by multi-view clustering with enhanced consensus

**DOI:** 10.1186/s12920-017-0306-x

**Published:** 2017-12-21

**Authors:** Menglan Cai, Limin Li

**Affiliations:** School of Mathematics and Statistics, Xi’an Jiaotong University, Xianning West 28, Xi’an, China

**Keywords:** Subtype identification, Multi-view clustering

## Abstract

**Background:**

The Cancer Genome Atlas (TCGA) has collected transcriptome, genome and epigenome information for over 20 cancers from thousands of patients. The availability of these diverse data types makes it necessary to combine these data to capture the heterogeneity of biological processes and phenotypes and further identify homogeneous subtypes for cancers such as breast cancer. Many multi-view clustering approaches are proposed to discover clusters across different data types. The problem is challenging when different data types show poor agreement of clustering structure.

**Results:**

In this work, we first propose a multi-view clustering approach with consensus (CMC), which tries to find consensus kernels among views by using Hilbert Schmidt Independence Criterion. To tackle the problem when poor agreement among views exists, we further propose a multi-view clustering approach with enhanced consensus (ECMC) to solve this problem by decomposing the kernel information in each view into a consensus part and a disagreement part. The consensus parts for different views are supposed to be similar, and the disagreement parts should be independent with the consensus parts. Both the CMC and ECMC models can be solved by alternative updating with semi-definite programming. Our experiments on both simulation datasets and real-world benchmark datasets show that ECMC model could achieve higher clustering accuracies than other state-of-art multi-view clustering approaches. We also apply the ECMC model to integrate mRNA expression, DNA methylation and microRNA (miRNA) expression data for five cancer data sets, and the survival analysis show that our ECMC model outperforms other methods when identifying cancer subtypes. By Fisher’s combination test method, we found that three computed subtypes roughly correspond to three known breast cancer subtypes including luminal B, HER2 and basal-like subtypes.

**Conclusion:**

Integrating heterogeneous TCGA datasets by our proposed multi-view clustering approach ECMC could effectively identify cancer subtypes.

## Background

Recent technologies have made it convenient to address medical and biological questions by using multiple and diverse genome-scale data sets. For example, The Cancer Genome Atlas (TCGA) has made a large-scale efforts to collect diverse types of genomic information from thousands of patients for over 20 cancers. To capture the heterogeneity of biological processes and phenotypes, integrative computational methods are needed to find the underlying data structure by combining all data types, which could help identify cancer subtypes. For example, [1] proposes a framework for joint modeling of discrete and continuous variables that arise from integrated genomic, epigenomic, and transcriptomic profiling which is applied on distinct integrated tumor subtypes discovery. In many other application domains, it is also commonplace that a single object can be described by multiple feature representations or *views*. For example, a webpage from the Internet can be represented by its text contents and the hyperlinks to the webpage, and a scientific publication can be represented by its text contents and citations. A better clustering result of samples is expected to be obtained if information from all views is taken into account. *Multi-view clustering* aims to combine multiple data information from different views to improve the clustering performance.

The challenge in multi-view learning is to efficiently reconcile the conflicting information among views. For the learning task with multiple views, the geometric distributions, similarity measurements and feature scales may vary a lot across different views. Samples represented in different views may have its own neighborhoods, density of distribution, magnitude, or noise process. The disagreement caused by these differences may hamper the clustering task.

Multi-view approaches can be roughly divided into the following two families. One is to learn an optimal linear combination of multiple kernels [2–12]. For example, optimized kernel k-means is proposed in [3] to find optimal linear combination of multiple kernels and an optimal cluster assignment matrix together by minimizing a trace clustering loss. The multiple kernel k-means clustering [6] is proposed to find the optimal combination coefficients of kernels by minimizing the clustering loss. Kernel k-means is then applied to the optimal combination of kernels. The second line is to determine low-dimensional projections by minimizing the differences or maximizing the correlations [13–19]. Other approaches propagate information from different views to construct graphs or similarities in a slightly different way. These methods include Multi-view EM [20], Multi-view spectral clustering [21, 22], Multi-view clustering with unsupervised feature selection [23, 24], Nonnegative Matrix Factorization [25], pattern fusion [26] and similarity network fusion [16]. For example, multi-view EM [20] takes the maximization and expectation in turn for different views, and the similarity network fusion (SNF) [16] fuses multiple networks to one network by iteratively updating a sequence of nonnegative status matrices.

However, all these methods assume that each view has a relatively large amount of information which favors the ground truth clustering structure. In other words, there exists a relatively strong signal of a common clustering structure across views. However, in real-world datasets, the common clustering structure information across views might be weak, while the disagreement among views might be strong. The varying degree of agreement and disagreement for each view might contaminate the underlying common clustering structure. Furthermore, certain views may contain subsets of features favoring different clustering structure. For example, in the clustering task for university webpages by text features, some words such as ‘major’, ‘position’ or ‘homework’ will lead to a partitioning of webpages into categories such as ‘student’, ‘faculty’ and ‘course’. However, the above clustering structure might be contaminated by other words (e.g. ‘biology’, ‘cell’, ‘computer science’, ‘code’ etc.), which might lead to a partitioning of webpages by their department of affiliation. We take another example of glioblastoma multiforme (GBM), an aggressive adult brain tumor. The integrative analyses based on different datasets often lead to conclusions including common and different parts. For example, one analysis [27] identified two subtypes by combining expression and copy-number-variant data, which does not agree with later findings in [28], which had identified four subtypes primarily by expression data. Interestingly, two subtypes found by [28] roughly correspond to the two subtypes identified in the work [29] by a DNA methylation-based approach, which also found a subtype related to somatic mutation in IDH1. Though methylation data was used in [28], the IDH subtype was not identified because the subtyping analysis was driven by the expression data.

In this work, we first propose a kernel-based multi-view clustering method with consensus (CMC), which aims to reconstruct kernels with a common clustering structure across views by maximizing the agreement among these kernels with preserving the similarity among original samples. The agreement between two kernels is measured by Hilbert Schmidt Independence Criterion (HSIC). To tackle the problem when different views show poor agreement, we further propose another multi-view clustering method with enhanced consensus (ECMC). The main idea of the ECMC model is to decompose each view into a consensus part and a disagreement part. The consensus parts for different views are supposed to be similar, and the disagreement parts should be independent with the consensus parts. Both of the two models can be efficiently solved by alternative updating with semi-definite programming. We apply our models to several simulation datasets, a publication dataset Cora and four Webkb datasets, and the results show that our ECMC model could achieve higher clustering accuracies than other state-of-art multi-view clustering approaches. We also apply the ECMC model to find cancer subtypes by combining mRNA expression, DNA methylation and microRNA (miRNA) expression data for five cancer data sets in TCGA, and the results show that our ECMC model outperforms other methods.

## Methods

### Problem statement

Suppose we are given a data set of *n* samples with *v* views, *X*={*X*_1_,*X*_2_,⋯,*X*_*v*_}, where $X_{i}\in \mathcal {R}^{p_{i}\times n}$(*i*=1,2,...,*v*) is the representation of data in the *i*-th view, and *n* is the number of observations. We assume that each $W_{i}\in \mathcal {R}^{n \times n}$ is a kernel computed by *X*_*i*_ for each *i*. We aim to do clustering on the *n* samples with the *v* multiple representations.

### Hilbert schmidt independence criterion

In this subsection, we introduce a measure of statistical independence which is called Hilbert-Schmidt Independence Criterion (HSIC) [30]. Intuitively, HSIC can be thought of as a squared correlation coefficient between two random variables *x* and *z* computed in feature spaces $\mathcal {F}{~}$ and $\mathcal {G}{~}$. Let *x* be a random variable from the domain $\mathcal {X}{~}$ and *z* be a random variable from the domain $\mathcal {Z}{~}$. Let $\mathcal {F}{~}$ and $\mathcal {G}{~}$ be feature spaces on $\mathcal {X}{~}$ and $\mathcal {Z}{~}$ with associated kernels $ k: \mathcal {X} \times \mathcal {X} \rightarrow \mathbb {R}$ and $l: \mathcal {Z} \times \mathcal {Z} \rightarrow \mathbb {R}$. If we draw pairs of samples (*x*,*z*) and (*x*′,*z*′) from *x* and *z* according to a joint probability distribution *p*_(*x*,*z*)_, then the Hilbert Schmidt Independence Criterion can be computed in terms of kernel functions via:

$$ \begin{array}{ll} \text{HSIC}(p_{(x,z)},\mathcal{F},\mathcal{G})&=\mathbf{E}_{x,x{\prime},z,z{\prime}}[k(x,x{\prime})l(z,z{\prime})]\\ &\quad+\mathbf{E}_{x,x{\prime}}[k(x,x{\prime})]\mathbf{E}_{z,z{\prime}}[l(z,z{\prime})] \notag \\ &\quad-2\mathbf{E}_{x,z}[\mathbf{E}_{x{\prime}}[k(x,x{\prime})]\mathbf{E}_{z{\prime}}[l(z,z{\prime})]], \end{array}  $$

where **E** is the expectation operator. The empirical estimator of HSIC for *m* points *X* and *Z* from *x* and *z* with *p*_(*x*,*z*)_ was shown in [30] to be

1 $$\begin{array}{*{20}l} \text{HSIC}((X,Z),\mathcal{F},\mathcal{G})\propto tr(KHLH), \end{array} $$

where *tr* is the trace of the products of the matrices, *H* is the centering matrix $H = I-\frac {ee^{T}}{m}$, *K* and *L* are the kernel matrices on the two random variables of size *m*×*m*. The larger HSIC, the more likely it is that *X* and *Z* are not independent from each other. HSIC can be considered as a similarity measurement between two kernels.

### Consensus multi-view clustering model (CMC model)

In the multi-view clustering problem, it is often the case that different views admit some degree of common underlying clustering structure of the data. Following a common idea of multi-view clustering approaches (e.g. [31]), we can also solve this problem by looking for clustering structures that are consistent across the views. Differently, our proposed CMC model for multi-view clustering aims to find new consensus kernels *K*_*i*_ for all the views by encouraging them to be similar or dependent across all the views. We also hope that the similarity information among samples in each view is preserved to some extent in the new kernel. HSIC is used as the similarity measurement between two kernels. Thus we propose the following CMC model:

2 $$ \begin{array}{c} \max\limits_{K_{1},\cdots,K_{v}} \sum\limits_{i} tr(W_{i}HK_{i}H) + \lambda \sum\limits_{i \neq j }{tr(K_{i}H K_{j}H)} \\ s.t.~K_{i} \geq 0, tr(K_{i})=1 ~~~~i = 1,\cdots,v, \end{array}  $$

where $H = I_{n}-\frac {ee^{T}}{n}$ is a centering matrix, *I*_*n*_ is an *n*×*n* identity matrix, and *e* is an *n*-dimensional column vector with all ones. The first term in the objective function makes sure the new consensus kernels preserve the original pairwise similarity information among samples for each view in the new consensus kernel, while The second term tries to maximize the agreement of the clustering information among different views. The semi-definite constraints of *K*_*i*_≥0 make sure *K*_*i*_s are kernels, and those of *t* *r*(*K*_*i*_)=1 make sure the objective function has upper bound. Once the reconstructed kernel for each view *K*_*i*_ is obtained, we could use spectral clustering by using a linear sum of *K*_*i*_.

However, the CMC model could not solve the problem when the common information among views are weak and disagreement information are strong. In this case, the ground truth clustering structure information in original *W*_*i*_ is too weak, and the ground truth consensus kernels *K*_*i*_ share little information with the original kernel *W*_*i*_. Thus it is very difficult to find the common clustering structure by encouraging to preserve original pairwise similarity information in the first term of the objective function. To tackle this problem, we further propose another kernel-based multi-view clustering model.

### Enhanced consensus multi-view clustering model (ECMC model)

To overcome the problem of poor agreement among views, we decompose each new reconstructed kernel *K*_*i*_ into two parts: a consensus part *C*_*i*_ and a disagreement part *D*_*i*_. We hope that the consensus parts *C*_*i*_s are similar across different views, while the disagreement parts *D*_*i*_s are far away from the consensus parts *C*_*i*_s. Thus we propose our enhanced consensus multi-view clustering model (ECMC) as follows

3 $$ \begin{array}{c} \max\limits_{\substack{C_{1}, \cdots, C_{v}, \\ D_{1}, \cdots, D_{v}}} \sum\limits_{i} tr(W_{i} H (C_{i}\,+\,D_{i}) H) +\alpha\sum\limits_{i\neq j}tr(C_{i} H C_{j} H)\,-\,\beta\sum\limits_{i,~j}tr(C_{i} H D_{j} H) \\ s.t.~ C_{i}, D_{i}\geq 0,~tr(C_{i})=1,~tr(D_{i})=1,~i=1, \cdots, v. \end{array}  $$

**Fig. 1:**
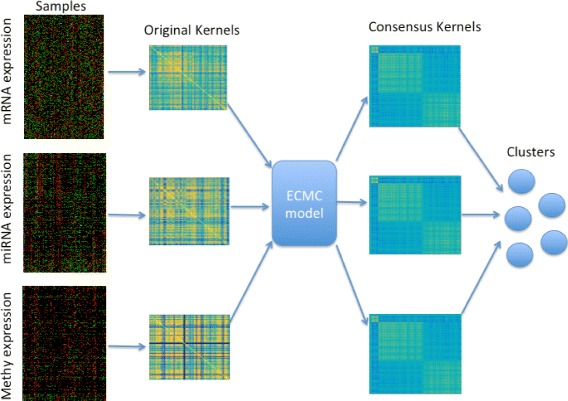
A flowchart of our ECMC approach for subtype identification

Different to our CMC model (2), we don’t encourage the similarity between the original kernel *W*_*i*_ and consensus kernel *C*_*i*_ any more. Alternatively, we encourage the similarity between *W*_*i*_ and the whole reconstructed kernel *K*_*i*_=*C*_*i*_+*D*_*i*_, which is more reasonable when there’s very weak common clustering information in *W*_*i*_. The second term in the objective function maximizes the similarity among consensus kernels, and the third term aims to make sure the consensus parts *C*_*i*_s are independent with the disagreement parts *D*_*i*_s as much as possible. The constraints are similar with the CMC model (2). By the ECMC model, we expect to throw away the disagreement information *D*_*i*_ from each view and keep the the consensus kernel *C*_*i*_ for the clustering task later on. The linear sum of consensus kernels *C*_*i*_s is finally used in spectral clustering for clustering the samples. Figure 1 shows the flowchart of our ECMC model.

With the computed *C*_*i*_ and *D*_*i*_, we define a consensus score

4 $$\begin{array}{*{20}l} \text{consensus}_{i} = \frac{tr(HK_{i}HC_{i})}{tr(HK_{i}H(C_{i}+D_{i}))}. \end{array} $$

to measure the amount of the consensus part in the *i*-th view. Note that the consensus score ranges from 0 and 1. If the score in one view is closed to one, it means the signals for the consensus part in the view are strong, and if it is closed to zero, it means that the disagreement part are dominant.

### Optimization algorithm

We apply the strategy of alternative updating to solve the optimization problems in both of the CMC model (2) and the ECMC model (3). We only discuss the optimization procedure for the ECMC model, and that for CMC model can be obtained in the same way.

We first fix *D*_1_,⋯,*D*_*v*_, and solve optimization problem (3) for optimal *C*_1_,⋯,*C*_*v*_ one by one. The *i*th optimization subproblem to solve for *C*_*i*_ can be written as

5 $$ \begin{array}{c} \max\limits_{C_{i}}\, tr(W_{i} H C_{i} H) +2 \alpha\sum\limits_{j\neq i}tr(C_{j} H C_{i} H) - \beta \sum\limits_{j}tr(C_{i} H D_{j} H)\\ s.t.~C_{i}\geq 0,~tr(C_{i})=1. \\ \end{array}  $$

By defining

6 $$ \begin{array}{c} M_{i}=H \left(W_{i}+2\alpha \sum\limits_{j\neq i} C_{j}-\beta\sum\limits_{j}D_{j}\right) H,\\ \end{array}  $$

the optimization problem in (5) is equivalent to

7 $$ \begin{array}{c} \max\limits_{C_{i}} ~~tr(M_{i} C_{i}) ~~~s.t.~C_{i}\geq 0,~tr(C_{i})=1. \\ \end{array}  $$

We then fix *C*_1_,⋯,*C*_*v*_ and solve the optimization problem in (3) for *D*_1_,⋯,*D*_*v*_ one by one. The *i*th subproblem can be written as

8 $$ \begin{array}{c} \max\limits_{D_{i}} ~tr(W_{i} H D_{i} H)-\beta\sum\limits_{j}tr(D_{i} H C_{j} H)\\ s.t.~~D_{i}\geq 0,~tr(D_{i})=1.\\ \end{array}  $$

It can be simplified as

9 $$ \begin{array}{c} \max\limits_{D_{i}} ~~tr(N_{i} D_{i}) ~~~s.t.~D_{i}\geq 0,~tr(D_{i})=1 \\ \end{array}  $$

with

10 $$ \begin{array}{c} N_{i} =H \left(W_{i}- \beta\sum\limits_{j}C_{j} \right)H. \end{array}  $$

The subproblems (7) and (9) are typical semi-definite programming problem, and can be solved efficiently by semi-definite programming toolbox CVX. The details of the procedure to solve ECMC model is presented in the ECMC algorithm box. In each outer iteration, line 4-line 7 is to update *C*_*i*_ one by one, using the current *D*_*j*_(*j*=1,⋯,*v*) and *C*_*j*_(*j*≠*i*), and line 8-line 11 is to update *D*_*i*_ one by one, using the current *C*_*j*_(*j*=1,⋯,*v*). The iteration stops when *C*_1_,⋯,*C*_*v*_ and *D*_1_,⋯,*D*_*v*_ converge with a small tolerance. In our experiments, we choose *W*_*i*_ - 2*I* and 2*I* as the initials for *C* *i* and *D*_*i*_ for each view, respectively.



## Results

### Measurements for clustering performance

We use the following two metrics to measure the clustering efficiency in the comparisons. The normalized mutual information (NMI) of a clustering ${\mathcal {C}} = \{C_{k}\}$ is defined as

$$\begin{array}{*{20}l} \text{NMI}({\mathcal{C}},{\mathcal{C}}^{*})=\frac{\mathrm{I}({\mathcal{C}},{\mathcal{C}}^{*})}{\sqrt{H({\mathcal{C}})\cdot H({\mathcal{C}}^{*})} }\quad \end{array} $$

with

$$\begin{array}{*{20}l} \mathrm{I}({\mathcal{C}},{\mathcal{C}}^{*}) = \sum\limits_{C_{k}\in {\mathcal{C}},C_{\ell}^{*} \in {\mathcal{C}}^{*}} p(C_{k},C_{\ell}^{*})\cdot \log_{2} \frac{p(C_{k},C_{\ell}^{*})}{p(C_{k})p(C_{\ell}^{*})}, \end{array} $$

where $H({\mathcal {C}}) = -\sum _{C_{i} \in {\mathcal {C}}}p(C_{i})\log _{2} (p(C_{i}))$, *p*(*C*_*k*_):=|*C*_*k*_|/*n*, ${\mathcal {C}}^{*}$ is the ground truth clustering, and $p(C_{i},C_{j}^{*})$ represents the joint probability of the two classes *C*_*i*_ and $C_{j}^{*}$. This can be estimated by the following formula [32]:

11 $$ \text{NMI}=\frac{\sum_{C,C^{*}}N_{C,C^{*}}\log\left(\frac{N\cdot N_{C,C^{*}}}{N_{C} N_{C^{*}}}\right)}{\sqrt{\left(\sum_{C} N_{C} \log \frac{N_{C}}{N}\right) \left(\sum_{C^{*}} N_{C^{*}} \log \frac{N_{C^{*}}}{N}\right) }},  $$

where *C* ^∗^ is a cluster in the true clustering assignment and *C* is a cluster in the computed clustering assignment, $\phantom {\dot {i}\!}N_{C}(N_{C^{*}})$ is the number of data objects in cluster *C*(*C* ^∗^), $\phantom {\dot {i}\!}N_{C,C^{*}}$ is the number of objects in cluster *C* as well as in cluster *C* ^∗^, *N* is the number of all the objects. NMI takes a value ranging from 0 to 1, and the closer to one it is, the more similar to true clusters the computed clusters are.

The other measurement is the average clustering accuracy (ACC) with the class labels {*l*_*j*_} of ${\mathcal {C}}$ in a suitable class ordering,

$$ ACC({\mathcal{C}},{\mathcal{C}}^{*}) = \frac1n \sum_{j=1}^{n} \delta(l_{j},l_{j}^{*}), $$

where the function $\delta (l_{j},l_{j}^{*})=1$ if $l_{j}=l_{j}^{*}$, or $\delta (l_{j},l_{j}^{*})=0$ otherwise.

For all the methods, we apply the normalized spectral clustering on the solutions of the compared algorithms. Since *k*-means in the last step of spectral clustering is sensitive to initials, 100 replications of *k*-means are performed using randomly selected initializations, and then the average clustering results are reported.

### Simulation study

#### Data simulation

We simulate several synthetic datasets to evaluate our proposed enhanced consensus model by comparing our methods with other state-of-art single-view and multi-view methods including spectral clustering on single views(SV1 and SV2), feature concatenation(Concat), co-regularized spectral clustering (Coreg) [15] and similarity network fusion (SNF) [16]. We generate the dataset of simulation 1 by the following procedure. We first generate 100 2-dimensional samples by a mixed Gaussian with different means of *μ*_1_=[−4 3]^*T*^ and *μ*_2_=[7 −8]^*T*^ and the same covariance matrix *Σ*_1_=[10 0 ; 0 5]. By adding white noises with strength 1, we could obtain two data matrices *A*_1_ and $A_{2}\in \mathcal {R}^{2\times 100}$. *A*_1_ and *A*_2_ have strong and similar clustering structure. We further obtain *B*_1_ and *B*_2_ by randomly permuting the samples in *A*_1_ and *A*_2_ and adding white noises again, respectively. After normalizing *A*_1_,*A*_2_,*B*_1_ and *B*_2_ such that each row has zero mean and 1 norm, we construct a matrix *X*_*i*_=[*A*_*i*_;*t* *B*_*i*_](*i*=1,2), where *A*_*i*_ and *B*_*i*_ is considered as the consensus part and the disagreement part, respectively. By changing the value of *t*, we can control the degree of disagreement in the dataset. We finally construct four datasets with *t*={0.95, 1, 1.2, 2} in simulation 1.

For simulation 2, we first generate *A*_1_ and *A*_2_ by another mixed Gaussian with means of *μ*_3_=[0 1]^*T*^ and *μ*_4_=[11 −10]^*T*^ and the same covariance matrix *Σ*_2_=[1 0 ; 0 1] with 100 samples. Different with the procedure in simulation 1, we generate *B*_1_ and *B*_2_ by randomly exchanging *s* samples from *A*_1_ and *A*_2_. Then we construct a matrix *X*_*i*_=[*A*_*i*_;*B*_*i*_](*i*=1,2). We control the degree of disagreement in the dataset by changing the value of *s*. We finally construct four datasets with *s*={25, 30, 40, 50} in simulation 2.

#### Experimental setting and results

We first compute a Gaussian kernel for each view and then apply all the comparison partners on the Gaussian kernels to obtain the clustering result. Note that k-means clustering is the final step for all these methods. Since it is prone to initials, we run 100 replicates of k-means and report the average result. For Coreg, CMC and ECMC methods, the parameters are all from the range of { 1*e*−10,⋯,1*e*+10}, and the best results are reported in Table 1.

**Table 1:** The average NMIs/ACCs and the standard errors obtained by the ECMC and other comparison partners in seven simulation data sets

	Methods	Simulation 1				Simulation 2			
		*t* = 0.95	*t* = 1	*t* = 1.2	*t* =2	*s* = 25	*s*= 30	*s* =40	*s* = 50
NMI	SV1	0.856	0.465	0.007	0.006	0.524	0.470	0.404	0.337
	SV2	0.775	0.495	0.012	0.002	0.524	0.470	0.421	0.331
	Concat	0.919	0.696	0.021	0.006	0.527	0.472	0.421	0.340
	Coreg	0.919	0.566	0.344	0.007	0.542	0.491	0.421	0.344
	SNF	0.960	**0.889**	0.012	0.005	0.562	0.510	0.421	0.519
	CMC	0.919	0.542	0.493	0.335	0.594	0.503	0.480	0.744
	ECMC	**1.000**	0.882	**1.000**	**1.000**	**0.667**	**1.000**	**0.859**	**1.000**
ACC	SV1	0.975	0.878	0.550	0.545	0.875	0.850	0.800	0.745
	SV2	0.960	0.886	0.565	0.525	0.875	0.850	0.800	0.750
	Concat	0.990	0.945	0.585	0.545	0.875	0.850	0.800	0.748
	Coreg	0.990	0.910	0.770	0.550	0.875	0.850	0.800	0.750
	SNF	0.995	**0.985**	0.565	0.540	0.875	0.850	0.800	0.780
	CMC	0.990	0.890	0.777	0.701	0.875	0.850	0.800	0.890
	ECMC	**1.000**	0.975	**1.000**	**1.000**	**0.898**	**1.000**	**0.980**	**1.000**

Highest NMIs/ACCs are marked in bold

We can see that in simulation 1, our proposed ECMC and SNF perform similarly with *t*=0.96 and 1. However our ECMC outperform when *t* is more than 1.This shows that when the consensus part is relatively weak, our method can also find the agreement information among all views. In simulation 2, we can find that, our method can always obtain the best NMI and ACC values.

**Fig. 2:**
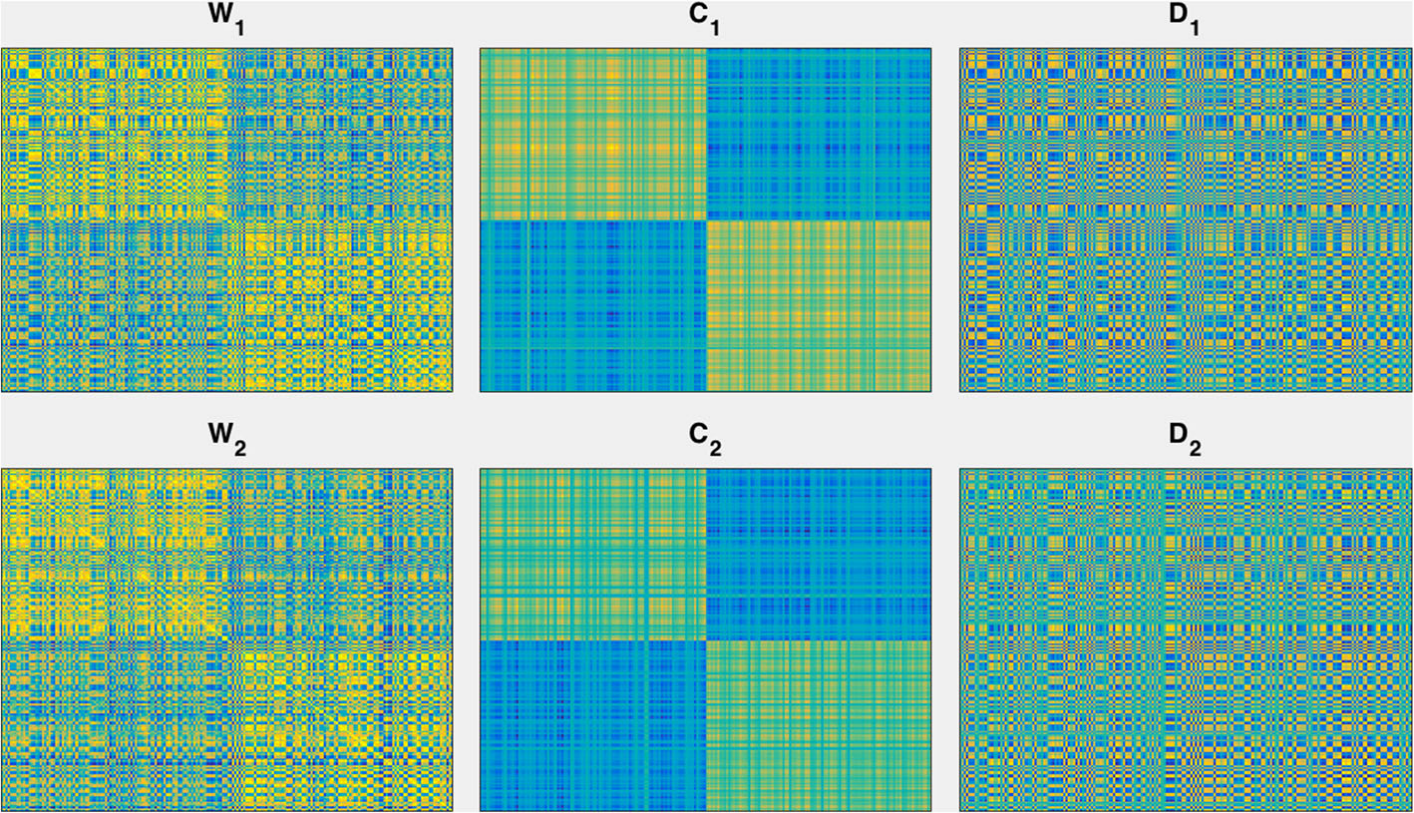
A demonstration of the ECMC model on a simulation dataset. *W*_*i*_s are given kernels from two views, and the ground truth clusters are the first half and the second half samples. *C*_*i*_s and *D*_*i*_s are obtained consensus kernels and disagreement kernels by our ECMC model, respectively. *C*_*i*_s have clear clustering structure consistent with ground truth labels

To further show the effectiveness of the ECMC model, we choose an example of *t*=2 in simulation 1. Figure 2 visualizes the original Gaussian kernels *W*_*i*_s, the computed consensus kernels *C*_*i*_s and the disagreement kernels *D*_*i*_s. From the figure, we can see that, the clustering structures in the original kernels *W*_*i*_s seem very weak, and the computed consensus kernels *C*_*i*_s have very clear clustering structures consistent with the ground truth.

### Benchmark machine learning datasets

We evaluate our approach on five benchmark machine learning datasets including four from Webkb datasets and one from Cora publication datasets.

#### Webkb webpage datasets

Webkb datasets consist of four sets of webpages from four universities Cornell, Texas, Washington, and Wisconsin, across five classes of course, project, student, faculty, and staff. Each webpage is represented by its text content and its hyperlinks. The class of staff, which has only a small number of samples, is removed. Table 2 lists the data summary for the datasets from the four universities. The datasets in each view are normalized such that each feature has zero mean and one norm.

**Table 2:** Summary of the real-world benchmark data sets: numbers of samples, features, views, and clusters

Data set		Cora	Cornell	Texas	Washington	Wisconsin
*#* of samples		397	91	102	156	179
*#* of features	view1	1,433	1,703	1,702	1,703	1,703
	view2	2,708	195	187	230	256
*#* of clusters		2	4	4	4	4

#### Cora publication datasets

The Cora dataset consists of 2708 scientific publications over seven categories (Neural Networks, Rule Learning, Reinforcement Learning, Probabilistic Methods, Theory, Genetic Algorithms, Case Based). Each publication is represented by two views. One is a 0/1-valued word vector indicating the absence/presence of the corresponding word from the dictionary which consists of 1433 unique words.The other is the citation relation with all other publications. We create a smaller subset with 397 publications which only consists of two categories of Rule Learning and Reinforcement Learning, and this dataset is used for evaluate our approaches. Similar to Webkb datasets, we also normalize the dataset for each view.

#### Experimental setting and results

For each dataset, we first compute Gaussian kernels for each view. We then compare our methods with the comparison partners by using these kernels. For Coreg, CMC and ECMC methods, the parameters are from the range of { 1*e*−10,⋯,1*e*+10}, and the best results are reported in Table 3. For SNF, we choose the size of neighbors *K* as the average cluster size and *η* from the set {0.3,⋯,1}, as suggested by the original paper. The best average clustering results over the parameters are reported.

**Table 3:** The average NMIs and ACCs and standard errors obtained by the ECMC and other comparison partners on real benchmark datasets

	Methods	Cora	Texas	Wisconsin	Washington	Cornell
NMI	SV1	0.021 ±0.001	0.175 ±0.001	0.273 ±0.004	0.252 ±0.001	0.182 ±0.002
	SV2	0.004 ±0.000	0.098 ±0.001	0.064 ±0.001	0.096 ±0.002	0.083 ±0.001
	Concat	0.002 ±0.000	0.120 ±0.001	0.120 ±0.001	0.128 ±0.001	0.156 ±0.001
	Coreg	0.025 ±0.001	0.234 ±0.002	0.284 ±0.005	0.306 ±0.002	0.213 ±0.005
	SNF	0.013 ±0.000	0.156 ±0.003	0.303 ±0.001	0.204 ±0.006	0.200 ±0.001
	CMC	0.085 ±0.003	0.316 ±0.002	0.343 ±0.003	0.328 ±0.002	0.326 ±0.002
	ECMC	**0.688 ±0.000**	**0.348 ±0.002**	**0.419 ±0.003**	**0.380 ±0.001**	**0.343 ±0.005**
ACC	SV1	0.587 ±0.003	0.570 ±0.001	0.533 ±0.004	0.440 ±0.001	0.456 ±0.004
	SV2	0.544 ±0.000	0.563 ±0.001	0.462 ±0.002	0.490 ±0.001	0.453 ±0.001
	Concat	0.511 ±0.001	0.383 ±0.003	0.375 ±0.003	0.375 ±0.002	0.411 ±0.001
	Coreg	0.590 ±0.001	**0.612 ±0.001**	0.558 ±0.004	0.519 ±0.003	0.496 ±0.005
	SNF	0.549 ±0.000	0.601 ±0.000	0.587 ±0.003	0.551 ±0.006	0.497 ±0.000
	CMC	0.665 ±0.004	0.468 ±0.003	0.578 ±0.005	0.492 ±0.002	0.479 ±0.002
	ECMC	**0.935 ±0.000**	0.566 ±0.001	**0.635 ±0.001**	**0.648 ±0.002**	**0.539 ±0.002**

The highest NMI and ACCs are marked in bold

**Fig. 3:**
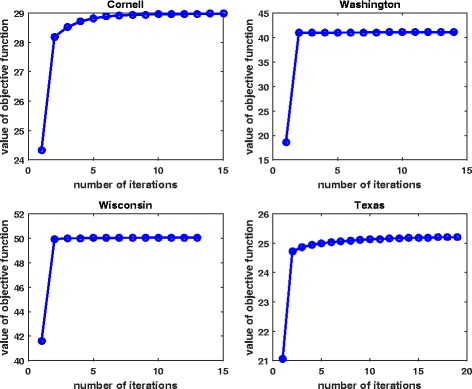
Convergence property of the ECMC by Webkb datasets

We report the average NMIs and ACCs for the benchmark datasets by all the methods in Table 3, respectively. From the table, we can see that, our proposed ECMC achieves the highest NMI values and ACC values among all the methods across all the five benchmark datasets, except that the Coreg obtains the highest ACC for the Texas data. Table 3 also shows that our CMC model could obtain the second highest NMIs among all the results. By using the measurement of ACC in Table 3, our CMC model is the second best for Cora data, and SNF performs the second best for all the four Webkb datasets. The results on the five benchmark datasets show the strong advantages of our ECMC model for clustering tasks. We also check the convergence property of our EMCM algorithm, and Fig. 3 shows that the algorithm converges after several iterations. We also compute the consensus scores of each view for each dataset. For Cornell data, the consensus scores of the two views are 0.141 and 0.896; For Washington data, the consensus scores are 0.212 and 0.049; the scores for Wisconsin data are 0.251 and 0.734; the scores for Texas data are 0.482 and 0.494. The consensus scores imply that each view may contain different amount of consensus information.

### Materials for subtype identification by TCGA data

We finally apply our ECMC model to identify cancer subtypes by conducting experiments on cancer genomics data from The Cancer Genome Atlas (TCGA) Research Network [33] for five cancer types: glioblastoma multiforme (GBM), kidney renal clear cell carcinoma (KRCCC), breast invasive carcinoma (BIC), colon adenocarcinoma (COAD) and lung squamous cell carcinoma (LSCC). The preprocessed data is provided by Wang et al. [16]. For each type of cancer, three data types are available: DNA methylation, mRNA expression and miRNA expression. We summarize the data in Table 4. For expression data, GBM and LSCC apply the Broad Institute HT-HG-U133A platform, BIC and COAD apply the UNC-Agilent-G4502A-07 platform, and KRCCC applies the UNC-Illumina-Hiseq-RNASeq platform. For miRNA expression data, BIC and GBM apply the BCGSC-Illumina-Hiseq-miRNAseq platform and the UNC-miRNA-8X15K platform,respectively, and LSCC, KRCCC and COAD use the BCGSC-Illumina-GA-miRNAseq. For the methylation data, GBM uses the JHU-USC-Illumina-DNA-Methylation platform, and BIC, LSCC, KRCCC and COAD apply the JHU-USC-Human-Methylation-27 platform. All the datasets also contain the clinical information including the overall survival data for patients. The problem of subtype identification aims to identify clusters where patients have a specific cancer subtype. Note that there’s no ground truth labels of subtypes for these datasets, and thus it is a discovery process.

**Table 4:** Data summary for the five TCGA cancer datasets

Cancer types	Patient number	mRNA expression	DNA Methylation	miRNA expression	Subtype number
GBM	215	12042	1491	534	3
BIC	105	17814	23094	1046	5
KRCCC	124	20532	24976	1046	3
LSCC	105	12042	27578	1046	4
COAD	92	17814	27578	705	3

### Clustering results

Two measurements, silhouette scores and Cox survival *p*-values, are used to evaluate the performance of our ECMC model for identifying subtypes for five cancers. Silhouette score [34] is used to measure the coherence of clusters by evaluating the similarity of patients within or between subtypes. Once we have the new representations for the samples and the subtype result for them, we could compute silhouette scores. The representations for different methods are different. For SNF and our ECMC, the new representations are obtained by spectral projection of the new kernels. For each sample *x*, let *m*_*x*_ represent the average dissimilarity for all samples in the same subtype and *n*_*x*_ represent the lowest average dissimilarity for all other samples in different subtypes. Euclidean distance is used to measure dissimilarity. The silhouette score for sample *x* is defined by *s*_*x*_=(*n*_*x*_−*m*_*x*_)/(*m* *a* *x*(*m*_*x*_,*n*_*x*_)). The silhouette ranges from -1 to 1. We compute the mean Silhouette value over all samples to measure how tightly all samples in the cluster are grouped. A silhouette score close to 1 implies a properly discovered clustering result. Another measurement is Cox survival *p*-values, which are computed using the Cox log-rank test [35] to measure whether the survival time is significantly different between the subtypes. For each sample, the survival time in months are given in the TCGA datasets. Lower Cox *p*-value implies that the survival profiles among subtypes are different more significantly, and thus the subtypes might be properly discovered.

For each cancer data, we first compute Gaussian kernel *W*_*i*_s for the three data types respectively, and then apply our ECMC model with *W*_*i*_s to reconstruct the consensus kernels *C*_*i*_ for each view. We finally do spectral clustering on the linear sum of these kernels $C=\sum \limits _{i}C_{i}$ to identify homogeneous cancer subtypes. The number of subtypes is chosen as 3, 5, 3, 4, and 3 following the work [16]. We also check the silhouette score with different number of clusters, and the results in Table 5 show that the selected number of clusters are reasonable since with them the silhouette scores achieve the highest or similar to the highest values. The parameter *α* is fixed as 10^10^, and *β* in ECMC model is chosen from the range of {10^8^,10^9^,10^10^} respectively. In Table 5, we report the silhouette scores with different *β* in this range, and we can see that for the five cancer types, the silhouette scores are relatively stable. For each combination of the parameters, we run 100 replicates of k-means and record the average silhouette score and the standard error.

**Table 5:** Silhouette scores for TCGA datasets for different parameters

	*k*								*β*		
S-score	3	4	5	6	7	8	9	10	10^8^	10^9^	10^10^
GBM	0.932	0.917	0.905	0.891	0.888	0.719	0.688	0.621	0.77	0.93	0.93
BIC	0.893	0.844	0.761	0.675	0.671	0.751	0.741	0.606	0.75	0.72	0.73
KRCCC	0.892	0.878	0.798	0.767	0.695	0.738	0.661	0.489	0.77	0.79	0.88
LSCC	0.874	0.845	0.813	0.784	0.684	0.648	0.621	0.630	0.72	0.84	0.72
COAD	0.791	0.729	0.547	0.459	0.463	0.465	0.465	0.465	0.57	0.79	0.68

We finally report the best average silhouette score in Table 6 over all the parameter combinations. We also report the average silhouette scores by single-view spectral clustering with the gauss kernel *W*_*i*_ for each of the three data types of mRNA expression, DNA Methylation and miRNA expression, respectively. The average silhouette scores are also reported in Table 6. We also apply the state-of-art multi-view clustering methods SNF and Coreg to the five cancer data sets. The experimental settings are similar with the ECMC model. The parameter *λ* in the Coreg method is chosen from the range of {10^−10^,⋯,10^10^}, and the parameters *K* and *η* in the SNF method are chosen from the ranges of {10,20,30} and {0.3,⋯,0.8}, as suggested in the original paper, respectively. The best average silhouette scores by the SNF and the Coreg over all their parameters are also reported in Table 6. From the results we can see that, our ECMC model can obtain highest Silhouette scores for all the five cancer data sets. This implies that the ECMC model is able to capture the clustering structure with tight clusters.

**Table 6:** Silhouette scores (S-scores) and Cox *p*-values obtained by different clustering methods

	Cancer types	mRNA expression	DNA Methylation	miRNA expression	Creg	SNF	ECMC
S-score	GBM	0.809 ±0.000	0.428 ±0.001	0.814 ±0.021	0.804 ±0.001	0.613 ±0.003	**0.930 ±0.000**
	BIC	0.254 ±0.001	0.318 ±0.002	0.468 ±0.003	0.310 ±0.002	0.526 ±0.002	**0.752 ±0.014**
	KRCCC	0.422 ±0.003	0.463 ±0.000	0.649 ±0.021	0.395 ±0.003	0.868 ±0.012	**0.889 ±0.000**
	LSCC	0.317 ±0.003	0.513 ±0.005	0.492 ±0.003	0.387 ±0.003	0.790 ±0.011	**0.844 ±0.013**
	COAD	0.449 ±0.000	0.470 ±0.005	0.555 ±0.001	0.468 ±0.000	0.684 ±0.005	0.793 ±0.000
*p*-value	GBM	0.805	0.563	0.188	8.40e-3	**2.85e-5**	3.12e-5
	BIC	1.22e-2	3.11e-3	0.216	3.26e-4	9.20e-5	**2.34e-7**
	KRCCC	1.16e-2	0.838	0.834	2.30e-3	8.71e-2	**1.98e-4**
	LSCC	1.10e-2	2.36e-2	0.572	1.90e-3	1.65e-4	**2.53e-4**
	COAD	0.171	8.53e-3	0.314	5.4e-3	1.20e-3	**9.34e-4**

The highest S-scores and lowest *p*-values are marked in bold

### Survival analysis

**Fig. 4:**
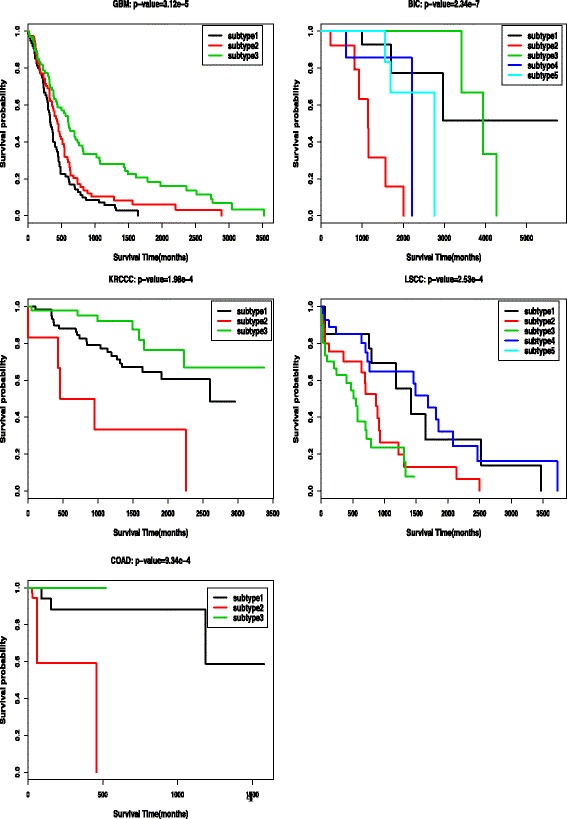
Kaplan-Meier survival curves for the five cancer types (*p*-values are reported in Table 6)

We further evaluate the performance of our ECMC model by survival analysis. Once a clustering result is obtained, we could conduct Cox log-rank test and compute the Cox *p*-values. In Table 6, we report the lowest *p*-values over all the possible parameters mentioned above for each method, respectively. We can see that, single data type analysis could not lead to significantly different survival profiles for most cases, while the ECMC model with multiple data types could achieve the most significant *p*-values for all the five cancer types, except for GBM cancer, the ECMC and SNF obtain similar significant levels. Figure 4 shows the Kaplan-Meier survival curves by the ECMC clustering result with most significant *p*-values for the five cancer types, where we could see the significant different survival profiles over different subtypes. In Table 7, we also report the consensus scores of the three views for the five cancers, corresponding to the clustering result with the most significant survival *p*-values. The results show that the average consensus scores are around 0.5, which implies that each view have half consensus information with others.

**Table 7:** Consensus scores in each view for the five TCGA cancer datasets

Cancer types	Gene expression	mRNA expression	DNA methylation
GBM	0.092	0.117	0.032
BIC	0.496	0.500	0.498
KRCCC	0.421	0.468	0.412
LSCC	0.405	0.175	0.291
COAD	0.491	0.500	0.491

**Fig. 5:**
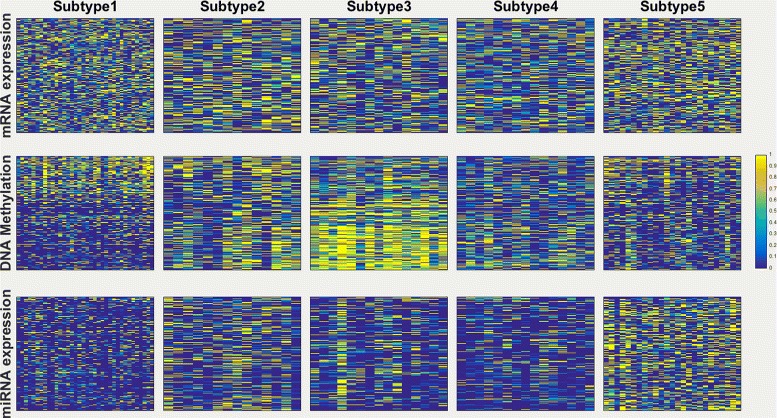
Visualization of the three data types in five subtypes for Breast cancer, with top row for mRNA expression, middle row for DNA methylation and bottom row for miRNA expression

Since the ECMC model could lead to the most significantly different survival profiles for the breast cancer data, we further analyze the obtained breast cancer subtypes. Figure 5 shows the visualization of the three views in five subtypes for Breast cancer. DNA Methylation has a very different profile among the five subtypes. Interestingly, Subtype 1 and Subtype 3 seem to have complementary DNA Methylation profiles. We also see that Subtype 1 and Subtype 5 have very different miRNA profiles as well. The combined signatures in mRNA, expression DNA methylation and miRNA expression data for the five subtypes are very different. We also compute the pairwise logrank *p*-values with Bonferroni correction, and found that Subtype 2 has significantly different survival profiles with Subtype 1, 3, 5 with corrected *p*-values of 1.16*e*−3,3.72*e*−4 and 1.88*e*−2.

**Fig. 6:**
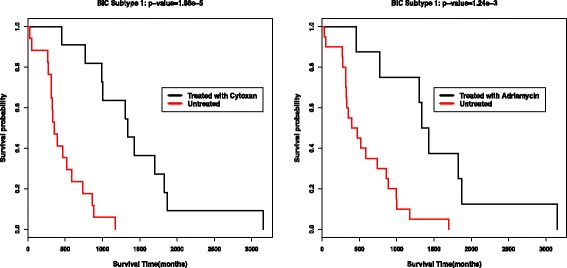
Survival analysis of the treatment with Cytoxan and Adriamycin in Breast cancer Subtype 1 (Cox log-rank *p*-value are 1.98e-5, and 1.24e-3 respectively)

**Fig. 7:**
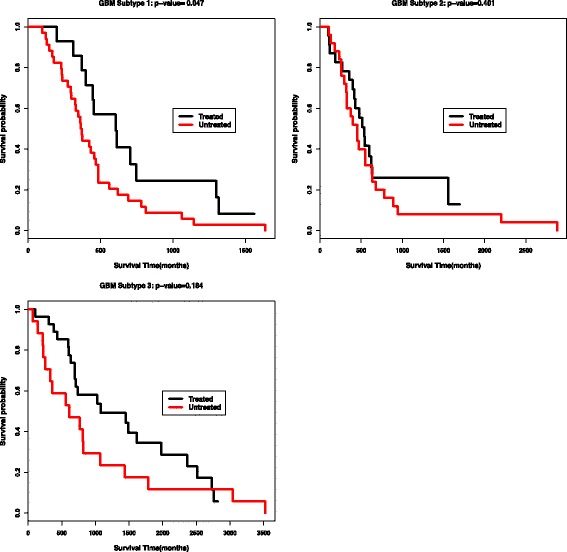
Survival analysis of the Temozolomide treatment in GBM subtypes. Significant *p*-value is obtained in Subtype 1(0.047) and no significant difference is obtained in the other subtypes

We finally conduct survival analysis to compare the survival profiles for finding interesting breast cancer subtypes. We choose three common treatments with drugs of Cytoxan, Adriamycin and Arimidex for breast cancers to do the analysis. For each treatment, survival analysis is conducted in all patients and also each subtype to compare the survival profiles between the patients with the treatment and the patients without the treatment. The computed Cox *p*-values for all treatments in all subtypes are reported in Table 8. The three treatments could not generate significantly different survival profiles between the treated patients and untreated patients from all the target population. However, in Subtype 1, and only in Subtype 1, both Cytoxan and Adriamycin could generate significantly improved treatment effects for treated patients, with *p*-values of 1.98*e*−5 and 1.24*e*−3. The Kaplan-Meier survival curves of these two treatments in Subtype 1 are shown in Fig. 6. In subtype 3, Arimidex could generate significantly improved treatment effects, with *p*-value of 1.82*e*−2. We also do the similar survival analysis for GBM cancer with treatment of Temozolomide. Figure 7 shows that the drug of Temozolomide could generate significantly improved survival profiles for GBM Subtype 1, and there’s no significantly difference in other two subtypes. This further shows that by our ECMC model, interesting subtypes could be discovered corresponding to different treatment effects.

**Table 8:** Survival analysis of three treatments on five BIC subtypes

Treatment	All	Subtype 1	Subtype 2	Subtype 3	Subtype 4	Subtype 5
Cytoxan	3.29e-2	**1.98e-5**	4.94e-2	0.310	0.447	0.226
Adriamycin	1.32e-2	**1.24e-3**	0.646	0.892	0.095	0.760
Arimidex	0.19	0.654	0.607	**1.82e-2**	0.433	0.352

The treatment could generate significantly improved treatment effects in the subtype of *p*-value in boldface

## Discussion

There are five known breast cancer subtypes including luminal A, luminal B, HER2-enriched, basal-like, normal-like [36]. Oestrogen receptor (ER), progesterone receptor (PgR), and HER2 are examined by usual immunohistochemical methods to define the subtypes as follows, luminal A subtype with ER and/or PgR (+), HER2 (-), luminal B subtype with ER and/or PgR (+) and HER2 (+), HER2 subtype with ER (-), PgR (-) and HER2 (+), basal-like subtype with ER (-), PgR (-) and HER2 (-), and unclassified subtype.

We first manually select some correlated genes for the basal-like breast cancer subtype. Curtis et al. [37] shows basal-like cancer enriched subgroup, harbours chromosome 5q deletions, and several signaling molecules, transcription factors and cell division genes were associated in trans with this deletion event in the basal cancers, including alterations in BUB1, CDCA4, CHEK1, FOXM1, HDAC2, KIFC1, MTHFD1L, RAD51AP1, TTK. Besides, [38] also found that loss of PTEN protein expression was significantly associated with the basal-like cancer subtype in both nonhereditary breast cancer and hereditary BRCA1-deficient breast cancer. Pires et al. [39] show alterations of EGFR, p53 and pTeN are cooperative and likely to play a causal role in basal-like breast cancer pathogenesis. These discoveries suggest that basal-like subtype may also correlate with the genes BRCA1 and EGFR, respectively. For each computed subtype (S1, for example) by our ECMC algorithm, We first calculate t-test *p*-values for each of these correlated gene to show whether the gene expression levels are significantly changed between the subtype S1 and the other subtypes. We then apply the Fisher’s combined probability test [40] to compute the group *p*-values for these genes, which could test whether the group of the selected genes are significantly different between subtype S1 and other subtypes. We do the same computation for each computed subtype S1 to S5, and report the results in Table 9. The results show that, our computed Subtype 1 is highly likely to correspond to the basal-like breast cancer subtype, with group *p*-value being 7.99e-6. Note that the treatment with Cytoxan and Adriamycin in Subtype 1 significantly extend the survival time, as shown in Fig. 6. It implies that these two drugs might be effective specially for basal-like breast cancer. Our computed Subtype 2 may also contains the basal-like breast cancer subtype, with group *p*-value being 2.03e-5.

**Table 9:** Group *p*-values for three breast cancer subtypes including luminal B, HER2 and basal-like

Group *p*-values	Subtype 1	Subtype 2	Subtype 3	Subtype 4	Subtype 5
luminal B	2.35e-01	**2.16e-13**	1.33e-02	5.03e-04	4.68e-02
HER2	3.87e-01	1.90e-02	3.34e-02	**3.18e-03**	2.65e-01
basal-like	**7.99e-06**	2.03e-05	4.53e-01	2.91e-03	4.40e-01

The subtype we found out, *p*-value in boldface, is likely to correspond to the true breast cancer subtype

We also manually select genes that are correlated with luminal B and HER2 breast cancer subtypes. For luminal B subtype, we include MAP2K4 since [37] show the recurrent deletion of MAP2K4 concomitant with outlying expression in predominantly ER-positive cases. PPP2R2A is likely to correlate with luminal B since [37] suggests the dysregulation of specific PPP2R2A functions in luminal B breast cancers. The genes ZNF703 and DHRS2 are also included since [41] confirm ZNF703 as a luminal B specific driver and Tumors with elevated ZNF703 levels were characterized by alterations in a lipid metabolism and detoxification pathway that include DHRS2 as a key signaling component. Curtis et al. [37] found ER-positive subgroup composed of 11q13/14 cis-acting luminal tumors which PAK1, RSF1 C11orf67, INTS4 reside in it. Loi et al. [42] found PIK3CA mutations are associated with low MTORC1 signaling and good prognosis with tamoxifen therapy in ER-positive which indicates PIK3CA have relation with luminal B subtype. Besides, ERBB2 is likely to correlate with HER2-enriched and luminal B subtypes, since the results in [37] show that HER2-enriched (ER-negative) cases and luminal (ER-positive) cases both belongs to ERBB2-amplified cancer. For HER2 breast cancer subtype, Pharmacologic FASN inhibitors were found to suppress p185(HER2) oncoprotein expression and tyrosine kinase activity in breast cancer overexpressing HER2 [43], which shows the correlation between FASN and HER2 type breast cancer. Bentires-Alj et al. [44] suggest that agents targeting GAB2 or GAB2-dependent pathways may be useful for treating breast tumors that overexpress HER2, and thus we include GAB2 as a correlated gene for HER2 type breast cancer. Besides, Trastuzumab blocks the HER2-HER3(ERBB3) interaction and is used to treat breast cancers with HER2 overexpression, although some of these cancers develop trastuzumab resistance. By using small interfering RNA (siRNA) to identify genes involved in trastuzumab resistance, [45] identified several kinases and phosphatases that were upregulated in trastuzumab-resistant cancers, including PPM1H. This suggests that PPM1H and ERBB3 may have some link with HER2 type breast cancer. With the manually selected gene sets for the two breast cancer subtypes, we also compute the group *p*-value for each computed subtype by our ECMC model. The results in Table 9 show that our Subtype 2 probably corresponds to the luminal B breast cancer type, with group *p*-value being 2.16e-13, and our Subtype 4 is likely to correspond to the HER2 breast cancer subtype.

## Conclusion

Our goal in this work is to discover consensus from different views when disagreement signals are very strong. We propose a novel decomposition strategy which tries to break down the information in each view into a consensus part and a disagreement part. The former parts are expected to be similar across all views for the sake of ‘consensus’, while the latter parts are expected to conflict with the consensus parts, for the sake of ‘disagreement’. The idea can be realized by making use of Hilbert Schmidt Independence Criterion, which could measure the similarities among kernels. Our ECMC model is proposed to reconstruct the consensus kernels and the disagreement kernels by maximizing the agreement among these kernels with preserving the similarity among original samples. Since consensus kernels are similar, the underlying clustering structure should be easy to be obtained. Our simulation experiments, real-world benchmark experiments and TCGA subtype identification experiments all show that the ECMC model outperforms other state-of-art multi-view clustering algorithms. In particular, we find some interesting subtypes in Breast cancer, and the survival analysis shows that the subtypes are significant. For the further research work, we will consider the following question. Although our ECMC model is effective for discovering consensus parts, it involves semi-definite programming which may be not as efficient as other computations such as eigenvalue decomposition in spectral clustering. We hope to formulate our idea in another way by avoiding semi-definite programming.
